# Cyclic transitions between higher order motifs underlie sustained asynchronous spiking in sparse recurrent networks

**DOI:** 10.1371/journal.pcbi.1007409

**Published:** 2020-09-30

**Authors:** Kyle Bojanek, Yuqing Zhu, Jason MacLean

**Affiliations:** 1 Committee on Computational Neuroscience, University of Chicago, Chicago, Illinois, United States of America; 2 Department of Neurobiology, University of Chicago, Chicago, Illinois, United States of America; 3 Grossman Institute for Neuroscience, Quantitative Biology and Human Behavior, Chicago, Illinois, United States of America; Ghent University, BELGIUM

## Abstract

A basic—yet nontrivial—function which neocortical circuitry must satisfy is the ability to maintain stable spiking activity over time. Stable neocortical activity is asynchronous, critical, and low rate, and these features of spiking dynamics contribute to efficient computation and optimal information propagation. However, it remains unclear how neocortex maintains this asynchronous spiking regime. Here we algorithmically construct spiking neural network models, each composed of 5000 neurons. Network construction synthesized topological statistics from neocortex with a set of objective functions identifying naturalistic low-rate, asynchronous, and critical activity. We find that simulations run on the same topology exhibit sustained asynchronous activity under certain sets of initial membrane voltages but truncated activity under others. Synchrony, rate, and criticality do not provide a full explanation of this dichotomy. Consequently, in order to achieve mechanistic understanding of sustained asynchronous activity, we summarized activity as functional graphs where edges between units are defined by pairwise spike dependencies. We then analyzed the intersection between functional edges and synaptic connectivity- i.e. recruitment networks. Higher-order patterns, such as triplet or triangle motifs, have been tied to cooperativity and integration. We find, over time in each sustained simulation, low-variance periodic transitions between isomorphic triangle motifs in the recruitment networks. We quantify the phenomenon as a Markov process and discover that if the network fails to engage this stereotyped regime of motif dominance “cycling”, spiking activity truncates early. Cycling of motif dominance generalized across manipulations of synaptic weights and topologies, demonstrating the robustness of this regime for maintenance of network activity. Our results point to the crucial role of excitatory higher-order patterns in sustaining asynchronous activity in sparse recurrent networks. They also provide a possible explanation why such connectivity and activity patterns have been prominently reported in neocortex.

## Author summary

Neocortical spiking activity tends to be low-rate and non-rhythmic, and to operate near the critical point of a phase transition. It remains unclear how this kind of spiking activity can be maintained within a neuronal network. Neurons are leaky and individual synaptic connections are sparse and weak, making the maintenance of an asynchronous regime a nontrivial problem. Higher order patterns involving more than two units abound in neocortex, and several lines of evidence suggest that they may be instrumental for brain function. For example, stable activity in vivo displays elevated clustering dominated by specific three-node (triplet) motifs. In this study we demonstrate a link between the maintenance of asynchronous activity and triplet motifs. We algorithmically build spiking neural network models to mimic the topology of neocortex and the spiking statistics that characterize wakefulness. We show that higher order coordination of synapses is always present during sustained asynchronous activity. Coordination takes the form of transitions in time between specific triangle motifs. These motifs summarize the way spikes traverse the underlying synaptic topology. The results of our model are consistent with numerous experimental observations, and their generalizability to other weakly and sparsely connected networks is predicted.

## Introduction

Network connectivity shapes dynamics in many systems and on many scales, ranging from gene transcription networks to epidemic spreading [1]. In the brain, neocortical architecture supports myriad complex functions. Before any of these functions can occur, neuronal spiking activity must be maintained throughout the lifespan of an animal. Stable maintenance of spiking activity—both as “background” activity and when tasked with inputs and outputs—is a basic function that arises from the structure of synaptic connectivity in the brain. Given the fact that the vast majority of excitatory synapses are weak and connections are sparse and recurrent, achieving stable activity is highly non-trivial [2–5]. Theoretical and experimental studies have characterized several architectural features that have the capacity to promote and shape spiking activity, such as a heavy-tailed synaptic weight distribution, excitatory clustering and the ratio between incoming and outgoing connections [3, 6–9]. Additionally, dynamical properties of ongoing activity, such as a balance between excitation and inhibition [8] and correlated spiking [10], are shaped by connectivity and in turn impact the continuation of spiking activity.

Neocortical activity in the awake mammal is characterized by low-rate, near-critical, and asynchronous dynamics. Any theory which purports to explain stable activity in neocortex must take these features of activity into consideration. Previous work has demonstrated that sustained activity co-occurs within a specific range of firing rates, supported by a balance between excitatory and inhibitory conductance [12, 19]. Firing rates that are too low or too high contribute to instability within the network [12]. Neocortex is also often characterized as having critical or near-critical dynamics [13]. In a practical sense, this entails activity which, in the absence of external input, is maintained without becoming epileptic nor dying out, and which follows a power law distribution in its active population size. The idea that neocortex operates near a critical point has a long history in neuroscience, going back to Alan Turing [14], and has been implicated in a number of desirable properties for neural networks [15]. For example, networks tuned near the critical point display maximum information transmission [13], information storage [16], and computational power [17]. Any incoming signals interact with and rely upon the activity state already present in a network, or the “background” activity, which in neocortex is generally asynchronous and irregular. Several theoretical studies have focused explicitly on self-sustained activity in the asynchronous state and the propagation of inputs in this state. They found a complex relationship between synaptic strength and firing rate in the maintenance of an asynchronous spiking regime [18–20]. Here we focus on said background activity and ask how it may be stably maintained in the absence of inputs. We build on previous theoretical and experimental studies by uncovering the dynamic mechanisms behind self-sustained activity using models that capture crucial factors of neocortical activity. Namely, the models match the ratio of excitatory to inhibitory units, the connectivity parameters, and the measurements of rate, synchrony, and criticality observed in neocortex.

Experimental results suggest that pairwise measurements alone may be insufficient to explain network dynamics. Higher-order patterns in both structure and activity have been reported to be intrinsic features of neocortex [21] and may be key to its function. Excitatory synaptic connectivity displays a prevalence of specific triplet motifs [2, 3] and cliques of neurons [8]. Activity in real neuronal networks exhibit elevated clustering [21–28] that is dominated by triplet motifs which can improve synaptic integration by coordinating the presynaptic pool [29]. Moreover, correlations between three units are necessary to recapitulate spatiotemporal spiking patterns [30]. Computationally, triplet motifs may improve coding [31, 32] and enhance perceptual accuracy and the prediction of responses in visual cortex [28, 33]. The causal relation from underlying synaptic connections to functional connections and correlations in activity within a network is complex [29, 34]. The presence of certain motifs in synaptic graphs greatly affects the strength of higher-order correlations in network neuronal activity [35]. Furthermore, the low firing rates and weak synaptic connections found in neocortex necessitate correlated inputs onto individual neurons [19, 36]. Here we build functional networks and then identify the intersection of the functional network with the synaptic network. We then analyze this subset of active synapses, or recruitment network, to study the dynamics that correspond to the activation of the underlying synaptic connectivity. Our focus is on the presence of higher order functional motifs and its relation with the maintenance of an asynchronous spiking state. First, a novel algorithmic approach is used to build large numbers of sparsely-connected recurrent spiking neural network models. Simulations are run on these models to explore the roles of realistic dynamics and higher-order interactions in sustained spiking activity. These models are recurrent and sparsely-connected; they are composed of excitatory and inhibitory adaptive exponential leaky integrate-and-fire (AdEx) neurons with conductance-based synapses [37]. Network topology parameters are varied and informed by connectivity seen in cortex [3, 6, 7]. By design, the models closely approximate both the statistics of connectivity as well as spiking activity in neocortex [20, 38, 39]. We find that simulations on the same network topology can either spontaneously stop (truncated run) or show sustained activity (sustained run), corresponding to different sets of initial membrane voltages. The dichotomy between sustained runs and this subset of late-truncating runs on the same networks, in addition to our ability to algorithmically construct and simulate very large numbers of networks, provided us with the opportunity to study the conditions which underlie sustained asynchronous activity.

## Results

### Network construction and simulation

**Fig 1:**
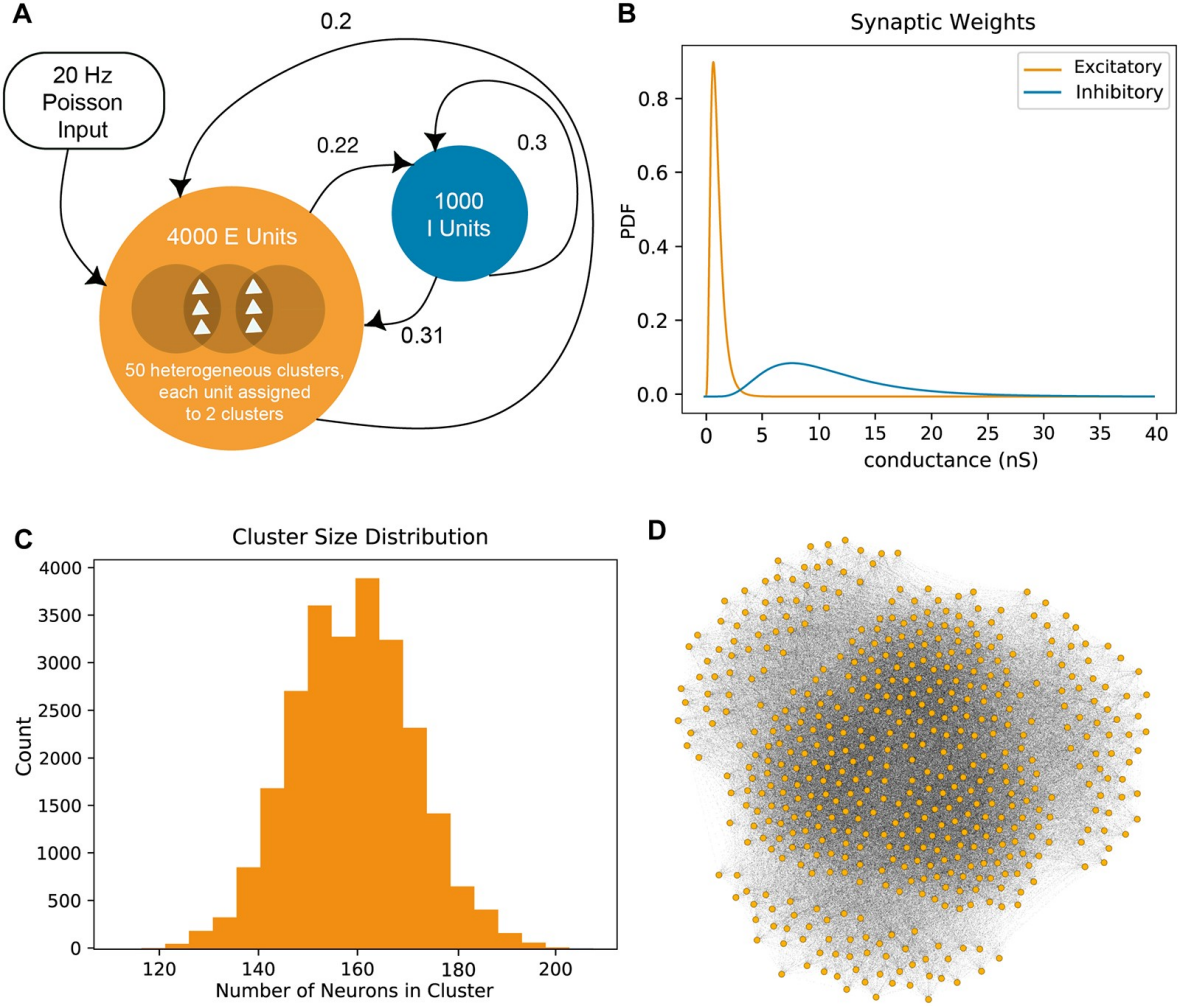
Network construction and search. A: Our networks were constructed with 4000 clustered excitatory and 1000 unclustered inhibitory units. Probabilities of connection (those from excitatory to excitatory units, and from inhibitory to inhibitory units) were inferred from experimental literature and determined via grid search (those from excitatory to inhibitory units and vice versa). Simulation runs began with 30ms of 20Hz Poisson input onto a subset of 500 units. B: Synaptic weights followed a log-normal (heavy-tailed) distribution. Synapses were conductance-based, so weights are in units of nanosiemens. Connections originating from inhibitory units were 10x stronger than those from excitatory units. C: For each network, we defined 50 clusters in total and randomly assigned each excitatory unit to two of these clusters. The wiring probability between units within the same cluster is twice that of units in different clusters. This resulted in heterogeneously-sized clusters. Here we show the cluster size distribution (in counts) for 500 networks. D: Visualization of a subset of 300 clustered excitatory units in our network.

Each spiking neuronal network was composed of 4000 excitatory and 1000 inhibitory adaptive exponential leaky integrate-and-fire (AdEx) units [37]. Synaptic connections were recurrent, sparse and conductance-based (Fig 1A). Excitatory connection strengths followed a heavy-tailed, log-normal distribution, where *μ* = −5.0*10^−5.0^ nS, *σ* = 0.5 nS, corresponding to a mean of 1.13 nS and a variance of 0.365 nS (Fig 1B). Networks therefore had a large number of weak connections and few strong excitatory synapses. Given that local connectivity in neocortex is clustered [2, 3]—although global statistical patterns of connectivity cannot be precisely determined from paired patch clamp recordings [40]—we also created clusters within our networks. For each network we defined 50 clusters and randomly assigned each excitatory unit to two of these clusters. Intra-cluster connection probability was twice that of inter-cluster connection probability. This resulted in excitatory clusters of different sizes (mean = 158.40, std = 12.27 units per cluster) [8]. The excitatory subgraphs had an average connection density (ratio of the actual over the total possible number of connections) of 0.211 (std: 1.10*10^−4^), of which 22.4% were reciprocal (std: 0.02%). Total density within each cluster was 0.389 (std: 3.11*10^−3^) and density between units in different clusters was 0.196 (std: 9.94*10^−5^) (Fig 1C and 1D).

At the beginning of a simulation trial, or run, initial resting membrane voltages were randomly assigned from a uniform distribution of -60 to -50 mV across all units. Activity was then initiated by 30 ms of 20 Hz Poisson input onto a set of 500 randomly chosen excitatory units (Fig 1A).

### Algorithmically identifying networks for analysis

We focus our study exclusively on network simulations which displayed naturalistic spiking dynamics. In order to evaluate large numbers of networks for biological realism while minimizing sampling bias, models were constructed, simulated, and scored algorithmically. We restricted the search for viable topologies to a range of connection likelihoods bounded by experimental observations [29]. This should not be interpreted to suggest that these connection likelihoods are the only viable solution for realistic spiking activity—we did not comprehensively survey the range of possibilities.

**Fig 2:**
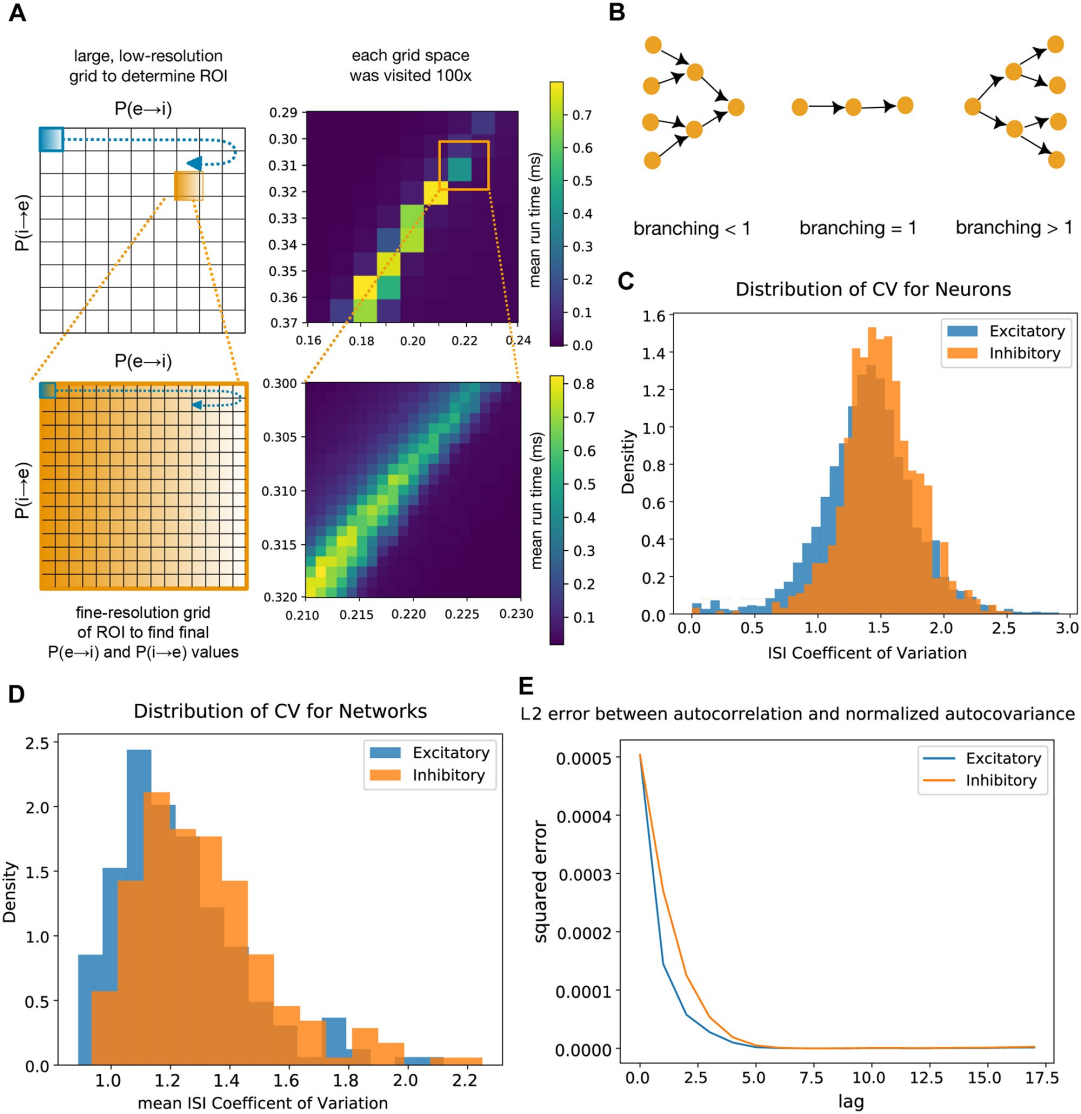
Grid search yields networks with dynamics resembling neocortex. A: We performed two rounds of grid search for the topological parameters that yielded consistent low-rate, critical, and asynchronous dynamics. The first search was at a lower resolution to narrow down our region of interest, and the second was at a finer resolution. B: One scoring metric we used was branching. The branching parameter [13] is a proxy for criticality. It measures the ratio of active descendant units to active ancestor units. A branching value of 1 indicates a balanced (or critical) network, which is the value we optimized for. C: Distribution of the interspike interval coefficient of variation for individual neurons. D: Distribution of the interspike interval coefficient of variation for networks. E: L2 error between autocorrelation and normalized autocovariance.

We identified viable topologies iteratively; in the first iteration, we performed a low resolution grid search for connection probabilities (Fig 2A). The values for the probabilities of connection from excitatory to excitatory units, *p*_*e*→*e*_, and from inhibitory to inhibitory units, *p*_*i*→*i*_, were inferred from experimentally measured connection probabilities in neocortex. They were 0.20 and 0.30 respectively [3, 29]. We performed grid search for the values of *p*_*e*→*i*_ and for *p*_*i*→*e*_. The existence of multiple classes of interneurons in neocortex means that this parameterization is a generalization—the values used for *p*_*i*→*i*_ and found for *p*_*e*→*i*_ and *p*_*i*→*e*_ represent summary values for a generic interneuron. During grid search, we rewired topologies within a limited range of *p*_*e*→*i*_ and *p*_*i*→*e*_, to identify sets of connection likelihoods that resulted in networks with sustained low-rate, near-critical, and asynchronous dynamics as observed in neocortex [20–33, 37–39, 41–44]. Criticality was measured using a branching parameter that is the ratio of active descendant units to active ancestor units across time [13]. A value of 1—where the number of active descendants is equal to the number of active ancestors—indicates critical dynamics (Fig 2B). We used a fast, on-line synchrony heuristic (variance of the mean voltage divided by the mean of voltage variances, see Methods) for the sake of grid search speed. A run was considered to be asynchronous if this heuristic value was below 0.5. Runs below this threshold correspond to a high mean Van Rossum distance which we employed throughout the remainder of the study [45, 46] (see Methods).

The first iteration of grid search isolated a region of interest, and we next used a higher resolution grid to find specific topologies each with the same probabilities of connection but differing in the specifics of connections (Fig 2A). To find these topologies we used the best results obtained from the second round of grid search, which were *p*_*e*→*i*_ = 0.22 and *p*_*i*→*e*_ = 0.31. These values were chosen for their ability to yield network simulations that had both low spiking rates and a high proportion of completion (see Methods).

These connectivity parameters were used to generate 2,761 synaptic topologies, where each unique topology is referred to as a network. For each network we randomly created 100 sets of input units, with 500 excitatory units per set. We ran 50 simulations on each set of input units, where each simulation began with different membrane voltages for all units. Each simulation lasted for as long as spiking activity was sustained, up to a maximum of 1 second. The spiking activity of each run on each network was then scored according to the average firing rate, the level of asynchrony, how balanced—or critical—the network was, and the duration of time over which spiking activity was maintained (see Methods). If a network’s average firing rate within excitatory units for all complete runs out of the initial 500 was less than 8 spikes/second, we added this network to the set of low-rate networks for subsequent analyses. High-rate networks were eliminated to maintain consistency with the low spike rates generally measured in neocortex. This yielded a final count of 87 low-rate networks. For each of these networks, we determined the set of input units which led to the most consistently sustained simulations, with the trade-off of rate increasing slightly. We will refer to these as a network’s optimal input units. Optimal input units were then fixed and used to generate 100 additional runs on each synaptic network; only the initial network state (i.e. membrane voltages of all units) varied. This generated a total of 8,700 unique runs, which we then analyzed.

To ensure that our simulations truly demonstrate sustained asynchronous irregular activity as seen in neocortex, we measured the distribution of the coefficient of variation of the interspike intervals of all neurons in every completed network simulation (Fig 2C), and also averaged across units within each completed simulation (Fig 2D). In both instances we find that networks are in an irregular regime (CV > 1). Averaging across networks we find a mean CV of 1.23 ± .21 for excitatory neurons and 1.31 ± .23 for inhibitory neurons. Furthermore, we measured the L2 error between the autocovariance normalized by variance and the autocorrelation of activity (after rate was no longer increasing in each trial) in both excitatory and inhibitory populations. The low level of difference seen between the two, and the rapidly decaying autocovariance, are qualitatively consistent with a stationary process (Fig 2E) [47]. Previous work has defined the asynchronous irregular state by using coefficients of variation greater than 1 to define irregularity and by using stable firing rate to define asynchrony [11, 19, 48]. Within this framework, our observations indicate our networks exhibit an asynchronous irregular regime with stationary activity.

**Fig 3:**
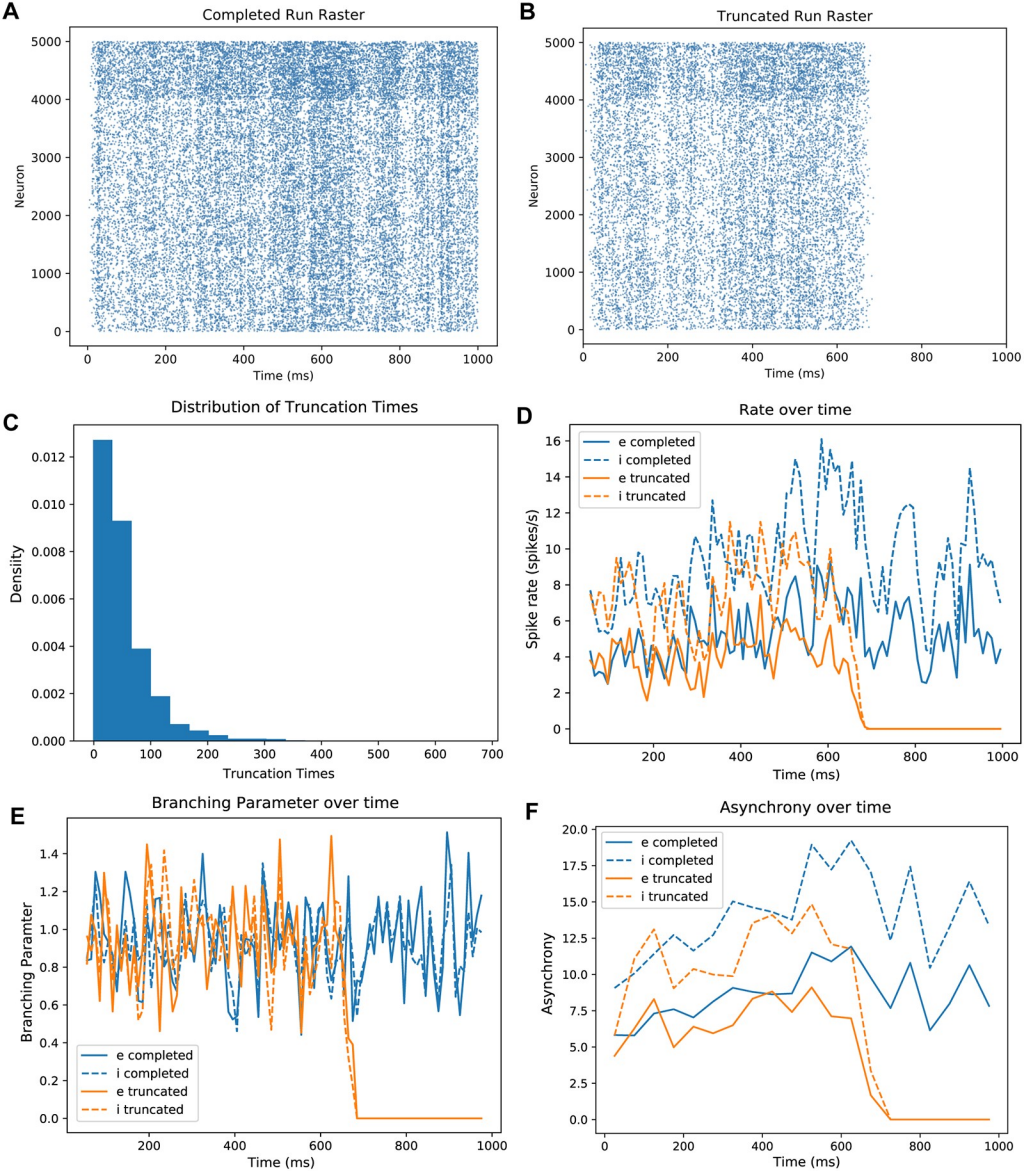
Simulations on the same network topologies yield sustained or truncated runs. A: A raster plot of a single complete 1000ms simulation on one of our networks. Excitatory units are numbered 1-4000 on the y axis, and inhibitory units are 4001-5000. B: A raster plot of a single truncated simulation (700ms) on the same network with the same input. C: Distribution of truncation times in ms for all truncated runs. D: Instantaneous rate across time for the simulations in rasters A and B, binned at 10 ms. Blue: sustained run; orange: truncated run; solid line: excitatory units; dashed line: inhibitory units. E: Instantaneous branching across time for the simulations in rasters A and B, binned at 10 ms. Same legend conventions as in D. F: Instantaneous Van Rossum distance across time for the simulations in rasters A and B, binned at 50 ms. Same legend conventions as in D.

### Rate, branching scores, and synchrony values on sustained and truncated simulations

We found that the same topology was capable of producing both sustained and truncated activity when only initial membrane voltages were varied. To be clear, the optimal input units and Poisson input trains were always the same but the membrane voltages varied. A run was sustained (or complete) if it displayed stable activity for the duration of a 1-second trial (Fig 3A). We found that all network simulations which reached 1 second were in each case able to sustain activity for the full duration of the simulation: we surveyed run times up to 10 seconds. We therefore chose one second as an indication of a network’s ability to sustain activity indefinitely, and as the definition of a successful run. If a network ceased all spiking before reaching the 1-second mark, that simulation was considered truncated (Fig 3B). Scoring analysis of the network spiking dynamics of rate, branching and synchrony between sustained and truncated run types revealed substantial overlap regardless of outcome.

**Fig 4:**
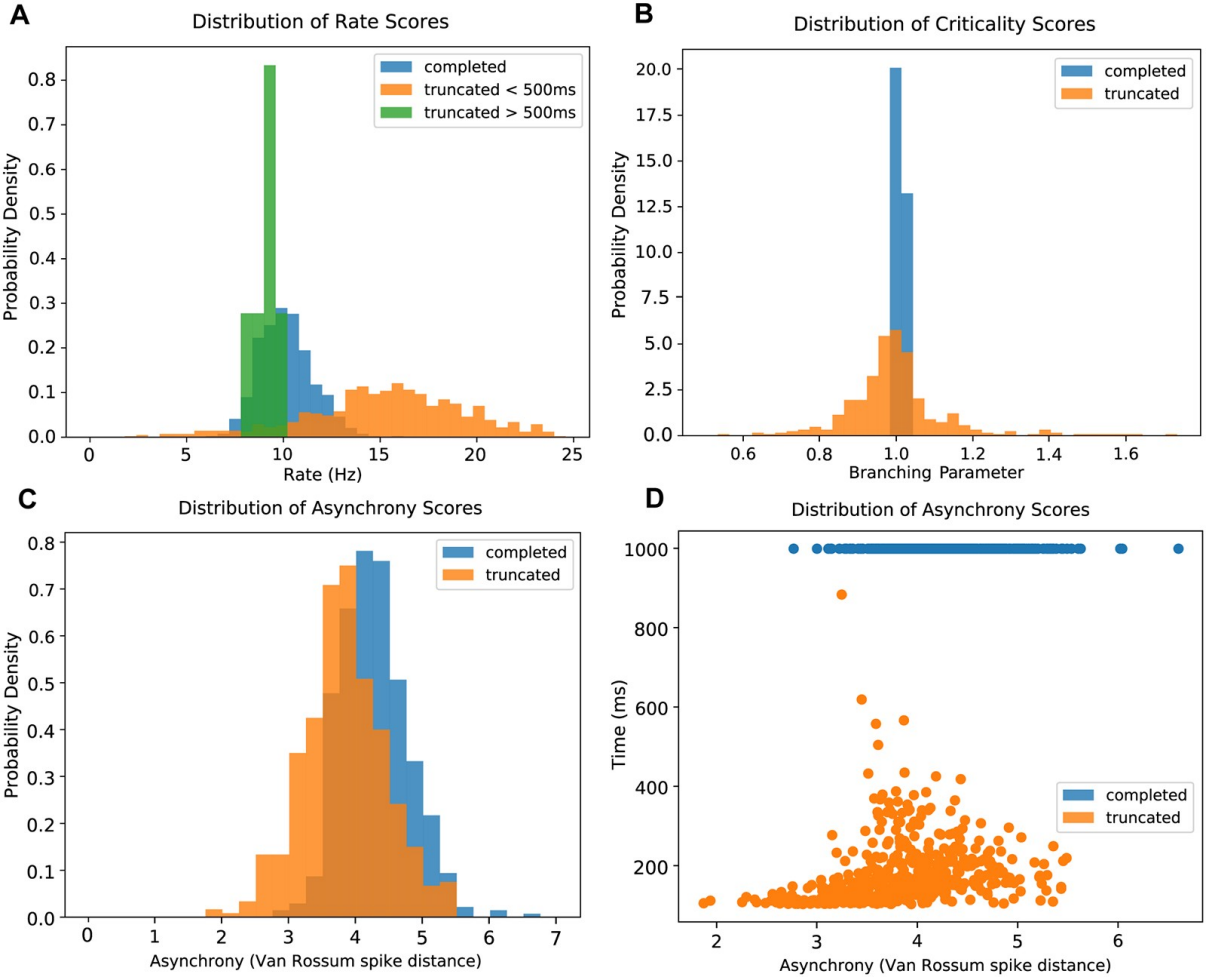
Score distributions for sustained and truncated runs. A: Distributions of spike rate scores for completed (blue), late-truncated (> 500 ms duration, green), and early-truncated (< 100 ms duration, orange) simulations. B: Distributions of criticality scores (branching parameter) for completed (blue) and truncated (orange) simulations. C: Distributions of asynchrony scores (Van Rossum spike distance) for completed and truncated simulations. D: Same asynchrony score data as in C, with completed and truncated simulations now separated along the y axis by their total durations. Each dot indicates an individual simulation.

Duration of truncated runs followed a long-tailed distribution, with the majority of runs truncating early (Fig 3C). Since activity within the excitatory units tended towards fewer spikes as a run approached truncation, we did not include the final 50ms of truncating runs in the calculation of rate, branching, or asynchrony scores. We also did not consider the stimulus period (initial 30ms), as we wished to analyze self-sustained network dynamics rather than stimulus-driven spikes. By focusing our analyses on the ‘middle portion’ of each run, we found that the rate, branching and synchrony values within excitatory units of both sustained and truncated run populations overlapped substantially. Runs that truncated within the epoch spanning 500ms to 990ms and sustained runs shared similar mean excitatory firing rates (9.77 and 10.14 spikes/s, respectively; *p* < .0005, *n* = 657, two sample chi square test). Runs that truncated between 140 and 400 ms had a significantly higher mean rate (15.65 spikes/s; *p* < 1 ⋅ 10^−15^, *n* = 1354, two sample chi square test), suggesting that higher firing rates contribute to instability of a network [12] (Fig 4A). The overlap index between rates of runs truncating later than 500 ms and sustained runs was 0.41 (95% CI 0.14 - .043). In runs that truncated earlier than 500 ms the overlap index with sustained runs was 0.27 (95% CI 0.23-0.30). Unlike rate, there was no difference in the scores between longer and shorter run times for both criticality and synchrony. The criticality score, measured using the branching parameter, was 0.997 ± 0.136 for truncated runs and 1.009 ± 0.004 for completed runs (*p* < 1 ⋅ 10^−15^, *n* = 979, two sample chi square test). The overlap index for criticality for the two run types was 0.31 (95% CI 0.21-0.35) (Fig 4B). Thus first order descriptors of rate and criticality within excitatory units, while different, substantially overlapped. This eliminated the possibility of a simple explanation of how and why activity was sustained in some cases while truncated in others.

As described above, only spikes from the middle portion of each run—that is, 30ms after trial start until 50ms prior to truncation—were used for the calculation of asynchrony scores. For the sake of computational efficiency during grid search, synchrony was defined as the variance of the mean voltage divided by the mean of voltage variances of all excitatory units (see Methods). Due to the dependence of this rapid measure upon voltages, and the fact that all voltages decay to resting potential after truncation, it will always yield high synchrony values for truncated runs. We therefore used Van Rossum spike distance for all excitatory units, normalized for run duration (see Methods) [45, 46], as our measure for each simulation’s asynchrony score outside of the initial grid search. As asynchrony increases, the Van Rossum distance also increases. The Van Rossum spike distance for our simulations was 3.82 ± 0.62 for truncated runs and 4.27 ± 0.51 for completed runs (*p* < 1 ⋅ 10^−15^, *n* = 1033, two sample chi square test). To provide some context, the corresponding Van Rossum spike distance of a rate- and size-matched network of uncorrelated Poisson units would be approximately 9. The overlap index for truncated and completed runs was 0.68 (95% CI 0.61-0.73) (Fig 4C and 4D). The values of Van Rossum spike distance were, like rate and criticality, highly overlapping between truncated and sustained runs. Thus rate, criticality, and synchrony levels substantially overlapped and did not cleanly partition truncated and sustained run types.

### Graph theory analysis of simulated networks

Having established that first order descriptions of network spiking failed to cleanly segment simulations into sustained and truncated runs, we next considered higher order descriptions. In previous work we have defined a taxonomy of active networks [29]. In that work we found that mechanistic insights underlying spiking network activity were provided by focusing on the subset of synaptic connections active during any one run. We refer to these networks as recruitment graphs and focus our analysis on these networks.

**Fig 5:**
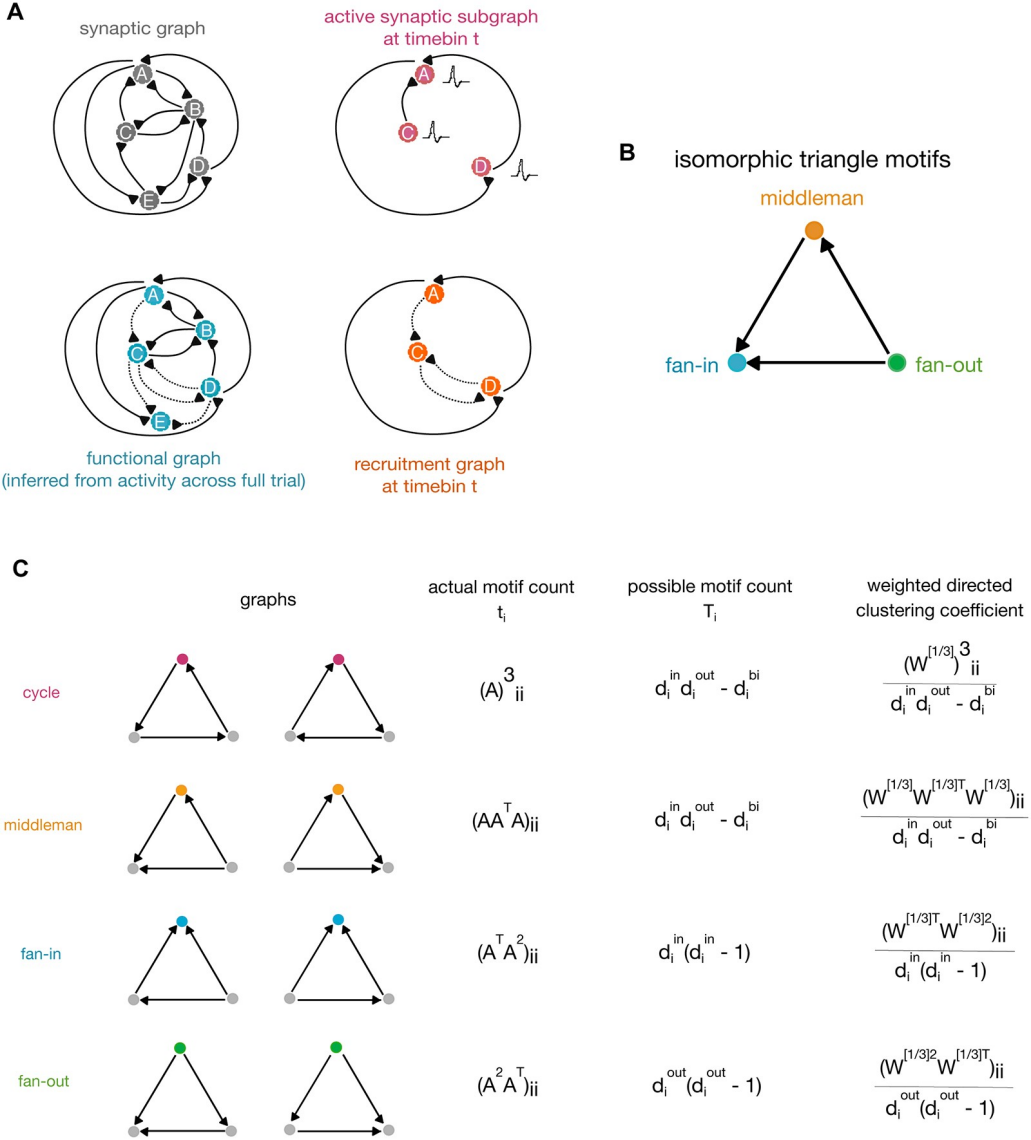
Graph and motif definitions. A: The synaptic graph is the ground-truth topology of our networks. Based on spiking activity during each simulation, we construct a series of active synaptic subgraphs—one for each time bin. These are graphs made of units which spiked in that bin, connected via the same edges as in the synaptic graph. We infer a single functional graph from whole-trial spiking activity using confluent mutual information—these graphs represent the functional connectivity of the network for each simulation trial. The intersection of the functional graph with the active subgraph for a given time bin yields the recruitment graph for that time bin. B: The three triangle motifs we examine—fan-in, fan-out, and middleman—are isomorphic by rotation. When calculating motif clustering, the choice of reference node is key. C: Calculation of the clustering coefficients of the different triangle motifs on weighted directed graphs, as defined in Fagiolo 2007 [49]. The clustering coefficient is defined as the ratio of the actual to the possible motif counts.

To do so, we begin with the structural connectivity matrix of our models as the synaptic graph (Fig 5A). We then constructed functional graphs using mutual information to quantify pairwise correlations between spiking neurons across each simulation. In order to generate a series of recruitment graphs, we identified the intersection of the functional graph with the synaptic subgraph according to the units which were active in each 10ms time step, resulting in one recruitment graph per time step. Weight values of functional and recruitment connections were calculated from mutual information and summarized in the functional graph, rather than taken from the synaptic weight matrix (see Methods). Due to our interest in the relationship between synaptic structure and functional spike maintenance, we focused our analysis on recruitment graphs. Thus the following results, unless otherwise noted, describe actual synaptic connections which have functional significance because they are active.

### Triplet motifs

The term ‘motif’ refers to a pattern formed by a group of units in a network. Previously we found that triplet motifs were informative of synaptic integration [29] and also increased the power of in vivo encoding models [28, 31–33]. Here we focused our analysis on similar patterns of connectivity in the recruitment network, involving groups of three units [49].

From the perspective of a single reference neuron, neighboring neurons can be arranged into four types of triplet motifs: fan-in, fan-out, middleman, and cycle. In isolating one triplet, the fan-in, fan-out, and middleman motifs are isomorphic by rotation, meaning that they only differ due to the choice of reference node (Fig 5B). The relative importance of a motif for a given neuron is measured by its contribution to that neuron’s clustering coefficient (Fig 5C). The clustering coefficient is the weighted ratio of the actual over the possible counts of a particular triplet motif type in which that neuron participates. Individual reference nodes in a given triplet may yield different clustering coefficients due to their specific weights and connections (see Methods).

It was possible that each of the algorithmically generated networks had different connection densities and weight distributions, which would impact weighted motif clustering coefficient measures. A measure that incorporated weight and controlled for density would be especially relevant since the recruitment graph density evolves over time. Furthermore, comparison with density matches is important given that sparseness itself results in enhanced small-world clustering [50]. We therefore used the measure of clustering propensity [51]. Propensity is the ratio of the clustering coefficients of the recruitment graphs compared to the average clustering coefficients of graphs with the same connection structure but randomly assigned connection weights. The propensity measure allowed us to compare different networks despite different connection densities and also allowed us to assess the impact of specific edge weights on triplet motif clustering coefficients [49]. A propensity value of 1 indicates that specific edge weights play a negligible role in clustering, since random edge weights would yield the same clustering coefficients (see Methods).

### Density and reciprocity statistics

**Fig 6:**
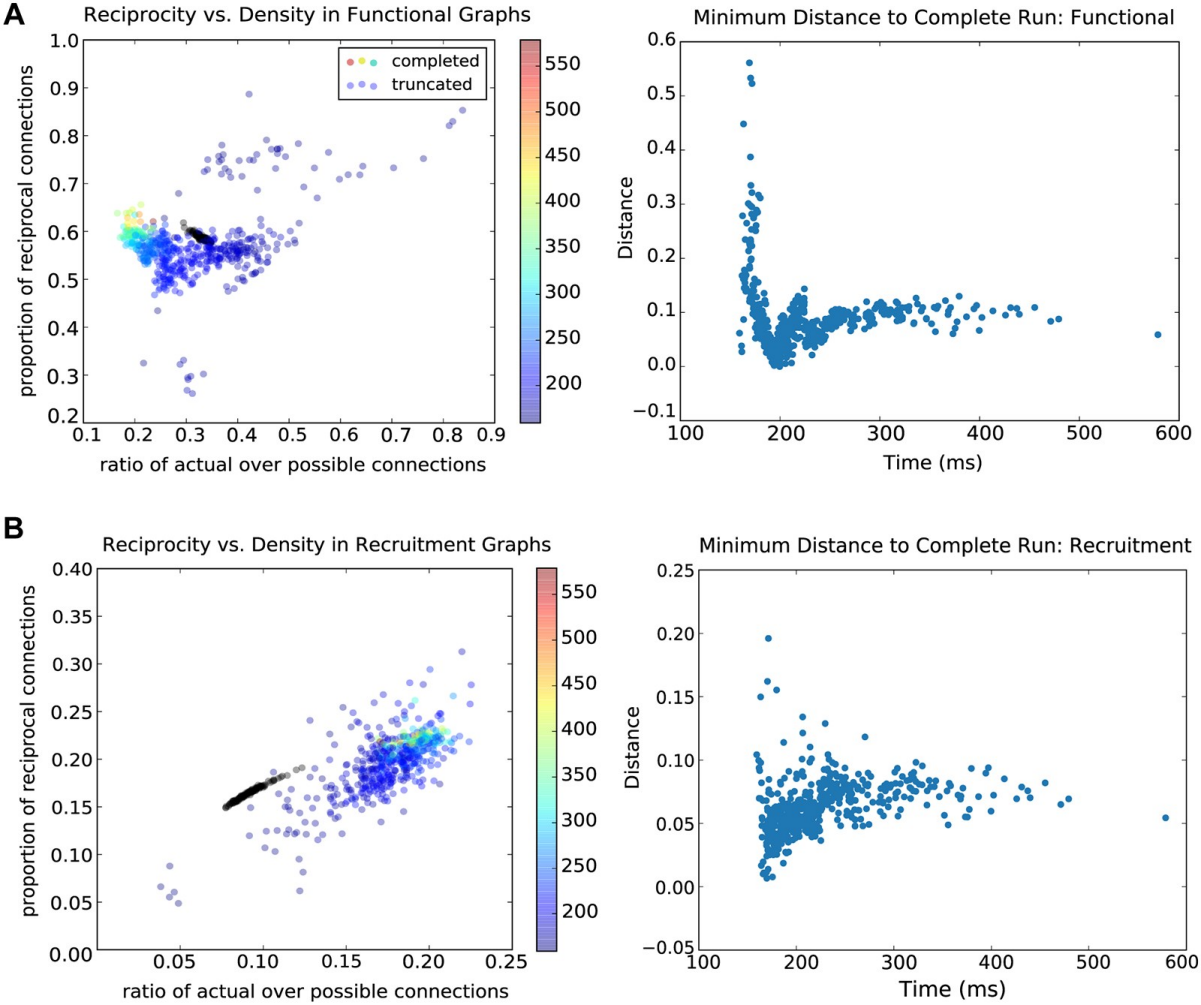
Standard network reciprocity. A: The left-hand panel shows the reciprocity (ratio of reciprocal connections to total connections) of **functional graphs** plotted as a function of their density (ratio of existing to possible connections). Data points for sustained runs are plotted in black and form a tight cluster, whereas those for truncated runs are varied. Truncated runs are colored by the ratio of inhibitory to excitatory spike rates. The right-hand panel shows the minimum distance (in reciprocity vs. density coordinate space for functional graphs) between each truncated run and a sustained run as a function of truncation time. Truncated runs which have greater than 200ms duration level off in their minimum distance. Thus, past a certain threshold, the difference between truncated and sustained runs’ density and reciprocity is not related to the run duration. B: The left-hand panel shows the reciprocity (ratio of reciprocal connections to total connections) of **recruitment graphs** plotted as a function of their density (ratio of existing to possible connections). Sustained runs are plotted in black and form a neat relationship between density and reciprocity and occur within a limited range of values. As in panel A, truncated runs are more diffuse. The color of each point indicates the ratio of inhibitory to excitatory spike rates. And also as in panel A, the right-hand panel shows the minimum distance between each truncated run and a sustained run (in reciprocity vs. density coordinate space) as a function of truncation time, this time for recruitment graphs. Truncated runs which have greater than 200ms duration level off in their minimum distance.

As reported above, synaptic networks were 21.1% connected, and 22.4% of connections were reciprocal. The functional networks of sustained runs, which were calculated using mutual information and were unique to each run, were more densely and also more reciprocally connected (Fig 6A, left). The functional networks averaged 32.6% (std: 0.6%) connectivity, of which 59.0% (std: 0.4%) were reciprocal. Recruitment graphs across time in sustained runs were sparser than the synaptic graphs, although only slightly less reciprocally connected (9.5% connected, std: 0.5%, and 16.7% recurrent, std: 0.5%) (Fig 6B, left). Functional and recruitment density and reciprocity did not differ significantly between sustained and truncated runs. However, there were more limited ranges for and a tighter relationship between density and reciprocity of both functional and recruitment graphs of sustained runs. In contrast, the spread of density and reciprocity, and of their relation, was more diffuse in truncated runs (Fig 6A and 6B). In addition to greater variance for truncated runs, there are differing trends in the variance, suggesting multiple modes of failure for a network simulation. The right-hand panels of 5A and 5B show, for functional and recruitment graphs respectively, the relationship between truncation time and the minimum distance between truncated and sustained runs, where distance is measured according to the 2D coordinate space of the left-hand panels (reciprocity vs. density). The minimum distance levels off for runs which exceed 200 ms in duration. Thus, the density and reciprocity measures of truncated runs do not approach those of sustained runs as duration increases. For runs which truncate prior to 200 ms, the minimum distances vary much more but are not strictly dependent on run duration. Thus the increased variance in truncated runs’ density and reciprocity compared to those of sustained runs is not dependent on run duration. This is particularly the case at time points beyond 100—200 ms.

### Triplet motifs in the different graph types

**Fig 7:**
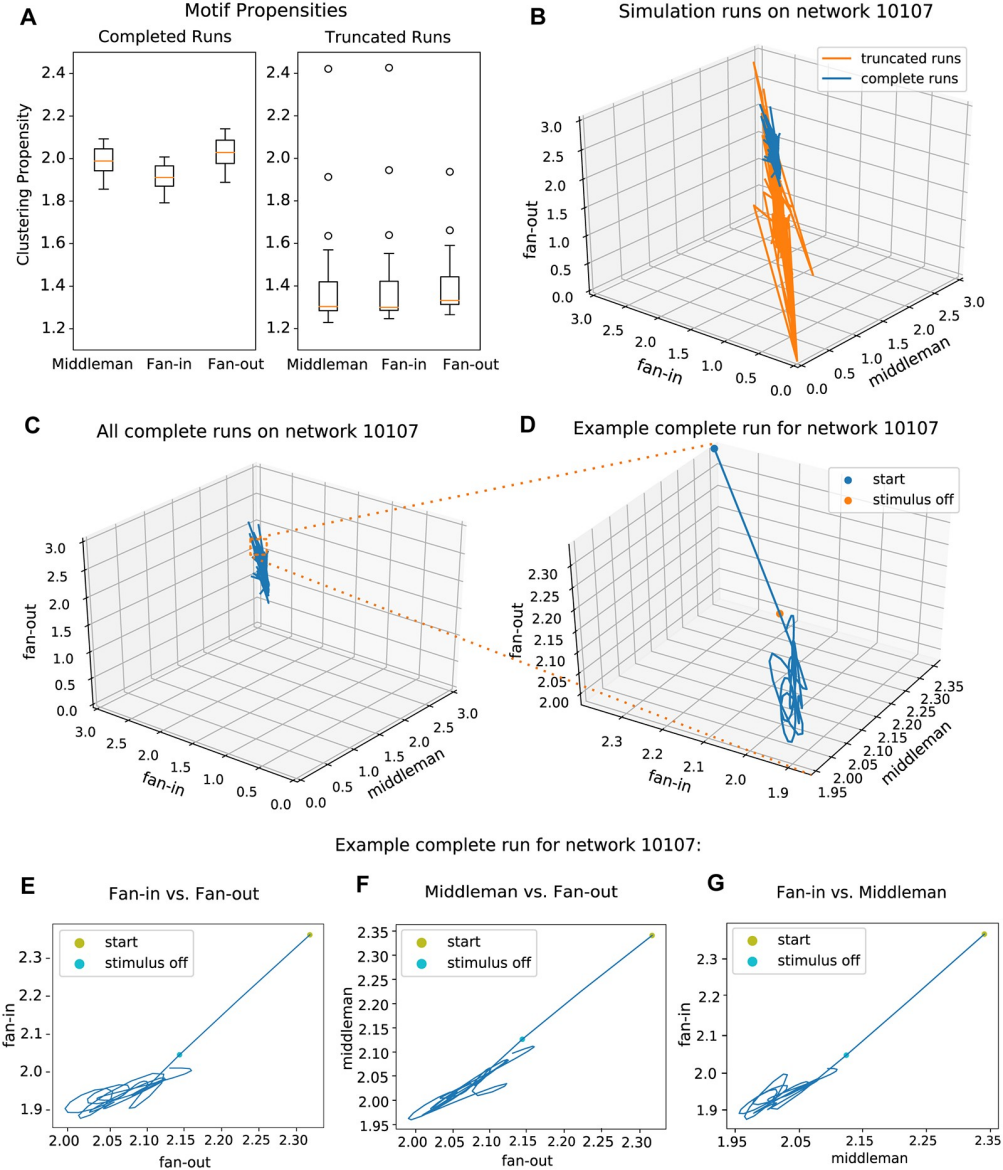
Standard network triplet motifs. A: Comparison of triangle motif clustering propensities of the three isomorphic motifs on sustained and truncated runs across all networks. B: Trajectories of all runs on a sample network in 3-dimensional isomorphic motif space. Truncated runs have a larger spread of trajectories along with variation in the ratios of inhibitory to excitatory spike rates. However, sustained runs are consistent in their spike rate ratios. C: Trajectories of all sustained runs alone, on axes of the identical scale as in panel B. D: Example trajectory of a single run on the same network, now enlarged (from inset in panel C). The network begins away from the area of its eventual cyclic trajectory, and the 30ms of Poisson input at the beginning of the run drives it towards this region. E: Example trajectory from panel D shown as fan-out propensity vs fan-in propensity. F: Example trajectory from panel D shown as fan-out propensity vs middleman propensity. G: Example trajectory from panel D shown as fan-in propensity vs middleman propensity.

We found that the three isomorphic motifs showed equal clustering in the synaptic graphs. This is expected of graphs with random, albeit clustered, synaptic connectivity. Clustering propensity centered at 1.00 (std = 7.8 * 10−5, 7.9 * 10−5, 8.0 * 10−5 for middleman, fan-in, and fan-out) for all three isomorphic motifs (Fig 7A). A value of 1 indicates that specific edge weights in synaptic graphs play a negligible role in clustering, since random edge weights would yield the same clustering coefficients. We found that in contrast to the static synaptic graph the dynamic functional and recruitment graphs were not random. The isomorphic motifs’ dominance in the recruitment graphs, or the strength of each motif’s contribution to overall clustering, varied over time in each trial. For sustained runs, motif clustering propensities for recruitment graphs (averaged across all time and all topologies) were 1.98 (std = 0.06), 1.91 (std = 0.06), and 2.03 (std = 0.07) for middleman, fan-in, and fan-out, respectively. Propensity values greater than 1, as these are, indicate that units in the recruitment graphs are more strongly clustered than would be expected in structurally-matched graphs with randomized weights. Motif clustering propensities also varied in recruitment graphs of truncated runs, with averages of 1.39 (std = 0.23), 1.40 (std = 0.23), and 1.43 (std = 0.24) for middleman, fan-in, and fan-out motifs (Fig 7A).

### Cycling of triplet motifs

To evaluate how the three isomorphic motifs co-varied across time for both successful and truncated trials, we plotted motif clustering propensities at each point in time against one another. We visualized this for all runs from a sample synaptic network (Fig 7B), and then examined sustained runs in particular (Fig 7C and 7D). Additionally, we colored the trajectory of each run according to the ratio of inhibitory to excitatory spike rates (Fig 7C). Truncated runs varied in the ratios of excitatory and inhibitory spike rates, consistent with the postulate that an imbalance between excitation and inhibition may have contributed to overall instability within the network. Sustained runs were consistent in their rate ratios. We further examined each two-dimensional projection of these motif transitions over time. 7E shows fan-in vs fan-out, 7F shows middleman vs fan-out, and 7G shows fan-in vs middleman for the complete run in (Fig 7D). As in the 3D case, clustering propensities formed a cyclic trajectory within a restricted region of 2D motif space. This indicates a systematic alternation between over- and under-representation of the three isomorphic motifs in the whole network relative to what would be expected in edge-matched networks. The cyclic trajectory within this region of motif space was consistent for all complete runs of low-rate, asynchronous, excitatory clustered networks we examined. We also found the same orderly temporal progression from one isomorphic motif to another when we considered clustering coefficient values as opposed to edge-normalized propensity. In contrast, truncated simulations were never restricted to this low variance cyclic alternation.

### Motif cycling and sustained activity

The motif cycling trajectory was not present at the moment of first spikes in a simulation. Rather, the path started at a point in motif space as determined by the initial membrane voltages of all neurons in the network. Injection of Poisson input drove network activity towards its eventual trajectory (Fig 7D, 7E, 7F and 7G). We identified two distinct types of truncation—in the first and far more common (97.7%) of the two, the simulation trajectory never approached or entered the region in propensity motif space where sustained runs lay. Truncation occurred rapidly after input stimulus ceased. In the second, rarer case (2.3%), the simulation successfully entered the sustained regime, yet after several hundred ms the trajectory destabilized, resulting in truncated activity. In this small subset of runs that exhibited trajectories prior to truncation, the trajectories did not follow a canonical path. Instead, motif dynamics during truncated runs transited in all directions away from the central region, demonstrating the multitude of ways in which activity structure can lose stability resulting in a failure of maintenance of an asynchronous spiking regime (Fig 7B). We examined whether the initial distance and input trajectory, which were determined by the initial conditions of the network and the Poisson stimulus, were determinants of successful activity maintenance. We found that even if the distance from the cycling region at the end of the stimulus period was minimal, some simulations still failed to enter into and stay within that regime. Others which were still distant from the region after the initial stimulus period continued on a successful trajectory and entered a stable cycling regime. These behaviors point to complex interactions between the network’s internal state and how input onto precise units within that network can influence maintenance of asynchronous spiking.

### Markov analysis

In order to quantify the cycling between isomorphic motifs, we constructed a Markov model for state transitions between dominant isomorphic motifs. We quantified cycling in this way because, while it does not give a sufficient account (the same markov chain could give different oscillatory behavior) of the observation, it does give us a necessary condition (a different markov chain could not generate the same observed pattern of oscillation). We described the network using a probabilistic voting scheme, as opposed to using analog propensity values. A unitary vote is cast by each unit for the motif type for which it has the highest propensity value at some time step. The proportion of total votes for each motif type is used to describe the relative dominance of that motif at that time step.

**Fig 8:**
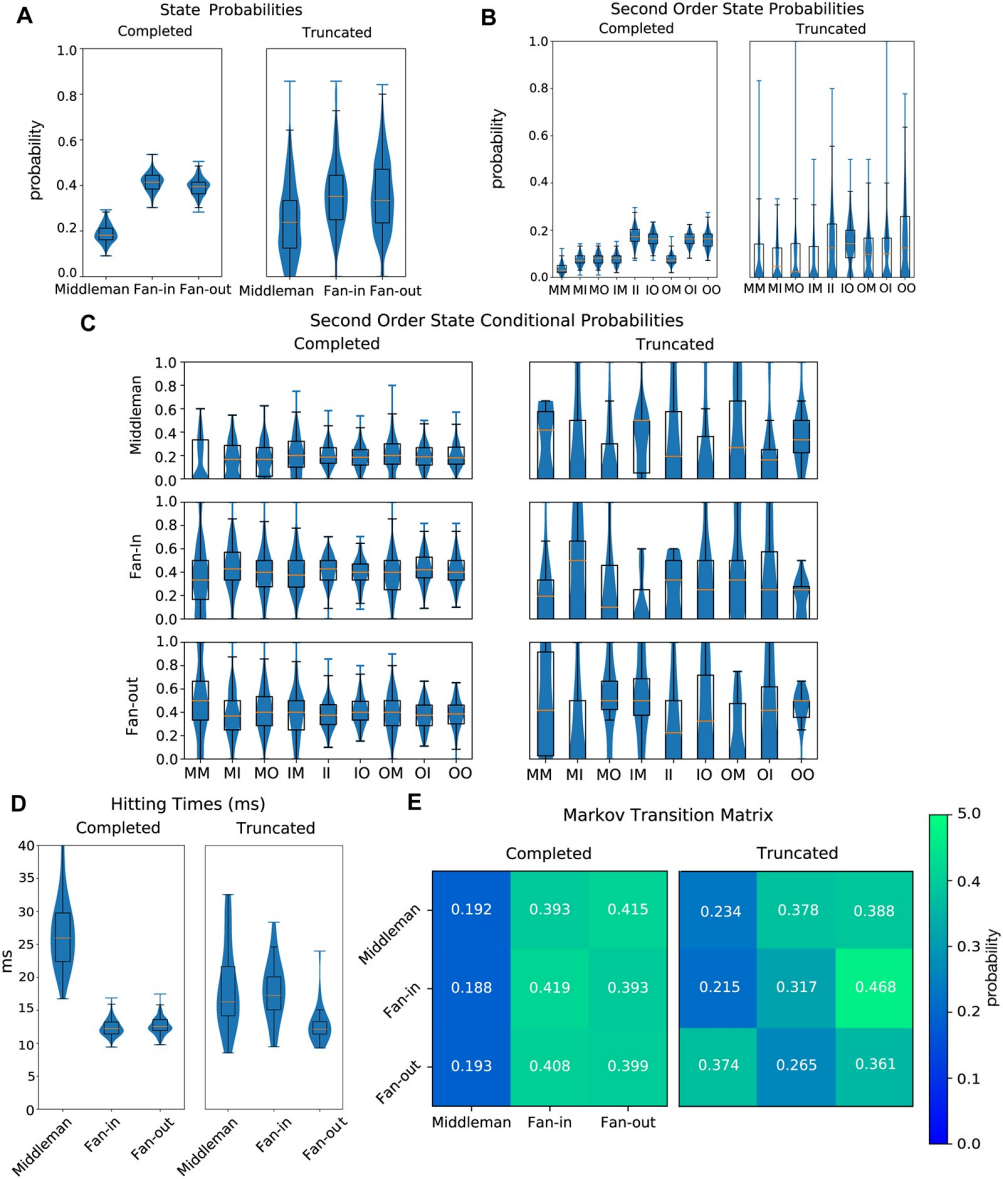
Markov comparisons between sustained and truncated runs on standard networks. A: Probabilities of state dominance of a triplet motif in sustained (left) and truncated (right) runs. B: Second order state probabilities for sustained (left) and truncated (right) runs. C: Second order conditional state probabilities for sustained (left) and truncated (right) runs. D: Expectation of hitting time for Markov model of state dominance transitions in sustained (left) and truncated (right) runs. E: Visualization of Markov matrix for state dominance in complete (left) and truncated (right) runs.

From this time series we constructed a Markov model transitioning between states. We found that the parameters characterizing the Markov process were canonical and low variance, such that successful cycling followed a specific reliable sequence between motifs. In contrast, the Markov parameters in simulations that truncated showed a failure to recruit this low-variance canonical sequence. First and second-order state probabilities and state transition probabilities significantly differed between sustained and truncated runs (*p* < 1 ⋅ 10^−15^, *n* = 1107, *p* < 1 ⋅ 10^−15^, *n* = 1107, *p* < 1 ⋅ 10^−15^, *n* = 884 respectively) (Fig 8A and 8B). Second-order state conditional probabilities also differed (*p* ≤ 0.029, *n* = 227) (Fig 8C). State probability is the probability of a motif being dominant at a given time. Second order probability is the probability with which a sequence of two motifs will be dominant at some given time. Conditional probability is defined as the probability of a motif given history of previous two motifs.

Markov analysis also gave the time scale which characterized motif cycling via the mean time for recurrence. This is defined by the expectation of the hitting time for each motif, given the network is currently dominated by that motif. We define hitting time, *t*, as *H*_*i*_ = inf{*n* ≥ 1 : *S*_*n*_ = *i*|*S*_*o*_ = *i*} and our expectation of hitting time, *t*, as $E\left[t\right]=\sum_{n=1}^{\infty }n\cdot p\left(H_{i}=n\right)$. This gives a mean recurrence time for each motif. We find truncating middleman to have mean 18.59 ms (std: 6.49 ms), completing middleman to have mean 27.02 ms (std 5.99 ms), truncating fan-in to have mean 18.01 ms (std: 4.61 ms) completing fan-in to have mean 12.39 ms (std: 1.27 ms), truncating fan-out to have mean 12.95 ms (std: 3.14 ms), and completing fan-out to have mean: 12.65 ms (std: 3.56 ms). Hitting times differed significantly between sustained and the small subset of truncated runs that entered this region of propensity (*p* ≤ 2.70 ⋅ 10^−6^, *n* = 227) (Fig 8D).

### Effects of connectivity weights

We hypothesized that the cycling between clustering propensities was necessary for sustained asynchronous activity due to the weak strength of the majority of individual synapses. Fan-in clustering has the highest probability of remaining in the state of fan-in clustering in the next time point which hints at the greater need for integration. But once integration is sufficient, the motif changes. For our model and for most of the synapses in neocortex, convergence of spikes from multiple sources must occur in order to evoke spikes in a receiving neuron [29]. Consequently we expected that as connection weights increased, the cycling between population-wide isomorphic motifs would lessen.

To test this, we strengthened all synaptic weights in the networks that previously scored well from 1.0x to 2.0x original values in increments of 0.1. Simulations were then re-run on these strengthened networks using the same stimulus and initial conditions. At 1.6 times the original weights, networks consistently displayed bursting activity. Consequently we restricted our analysis to networks with weights increased 1.5 times. All runs on these networks reached completion.

**Fig 9:**
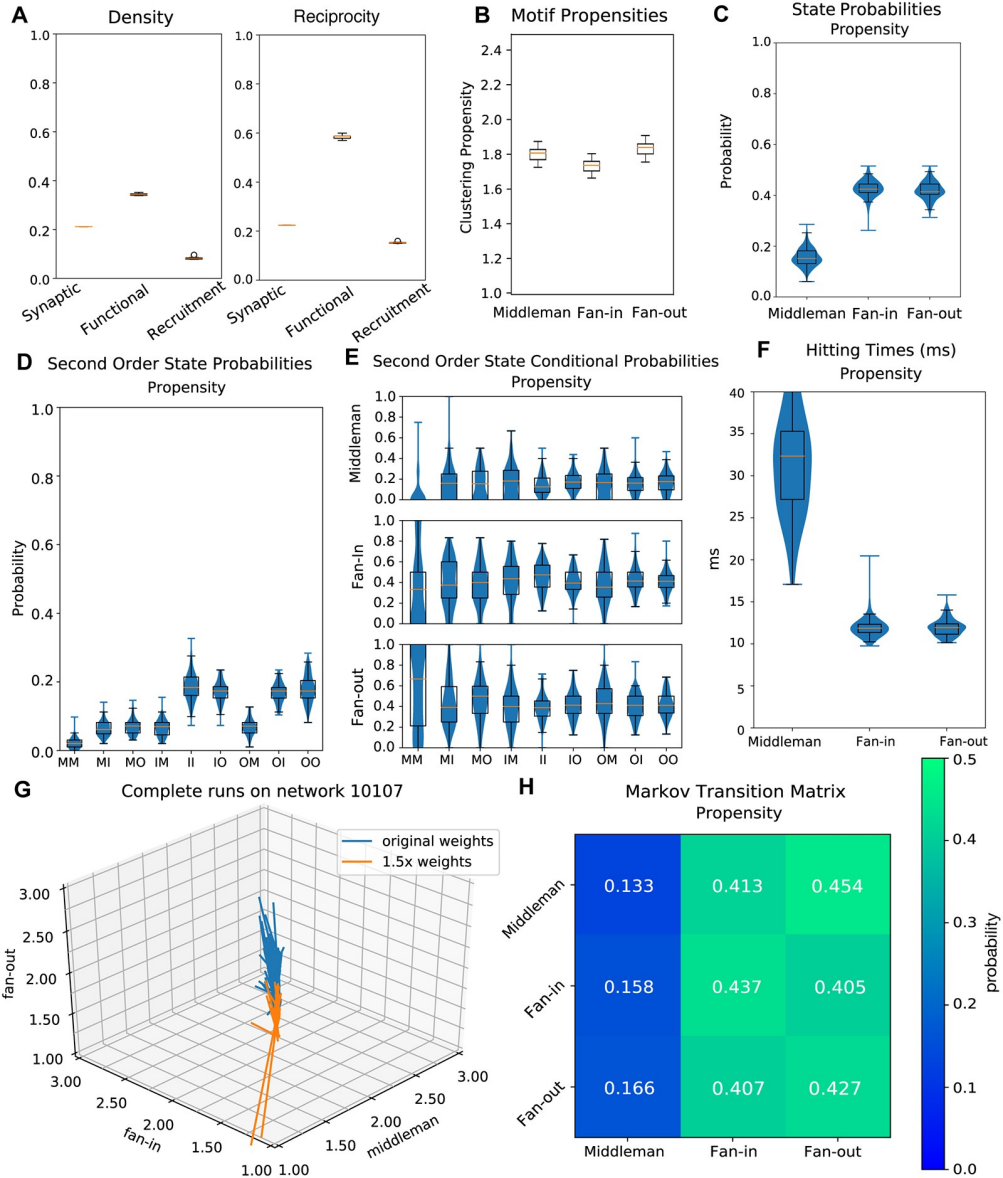
Networks with increased weights. Networks have the same structure as those seen in Figs 4 and 5, but all edge weights have been increased by 1.5 times their original values. A: Left, density (ratio of existing to possible connections) for synaptic, functional, and recruitment graphs. Right, reciprocity (ratio of reciprocal to all existing connections) for synaptic, functional, and recruitment graphs. B: Clustering propensity for isomorphic triangle motifs on increased-weight-graph simulations. The y-axis is scaled to match that of Fig 7A (clustering propensities on original graphs) and Fig 10B (clustering propensities on unclustered ER graphs). C: Probabilities of dominance of each triangle motif. The dominant motif at a time point is given by the maximum of mean middleman, mean fan-in, and mean fan-out across units. D: Second order motif state probabilities for progression of temporal recruitment graphs. E: Probabilities for each motif to follow a given second order motif. F: Hitting times for each state for the Markov process defined by motif transition probabilities. G: Trajectories of all complete runs on a sample network in 3-dimensional isomorphic motif space. In blue are the runs on the network with its original weights, in orange are the runs on the same network with weights increased. H: Markov Matrix for transition probabilities between motifs.

Increasing weights led to a decrease in all triangle motif propensities (Fig 9B), and also led to differences in the Markov characterization (Fig 9H). Motif state probabilities differed significantly between sustained runs on the original graphs and those on graphs with increased weights (*p* ≤ 0.00032, *n* = 296) (Fig 9C). Second-order state probabilities, state conditional probabilities, hitting times, and state transition probabilities all differed significantly as well (p < 0.01, p < 0.05, p < 0.005, p < 0.001, Fig 9D, 9E, 9F and 9H) (*p* ≤ 0.0065, *n* = 296, *p* ≤ .015, *n* = 267, *p* ≤ 0.0014, *n* = 267, *p* ≤ 0.011, *n* = 296 respectively), demonstrating the interaction of synaptic strength on the necessity of this regime (Fig 9). However the trend remained and in all sustained runs a low variance transition from motif to motif occurred.

### The dynamical motif solution is arrived at regardless of synaptic connectivity statistics

**Fig 10:**
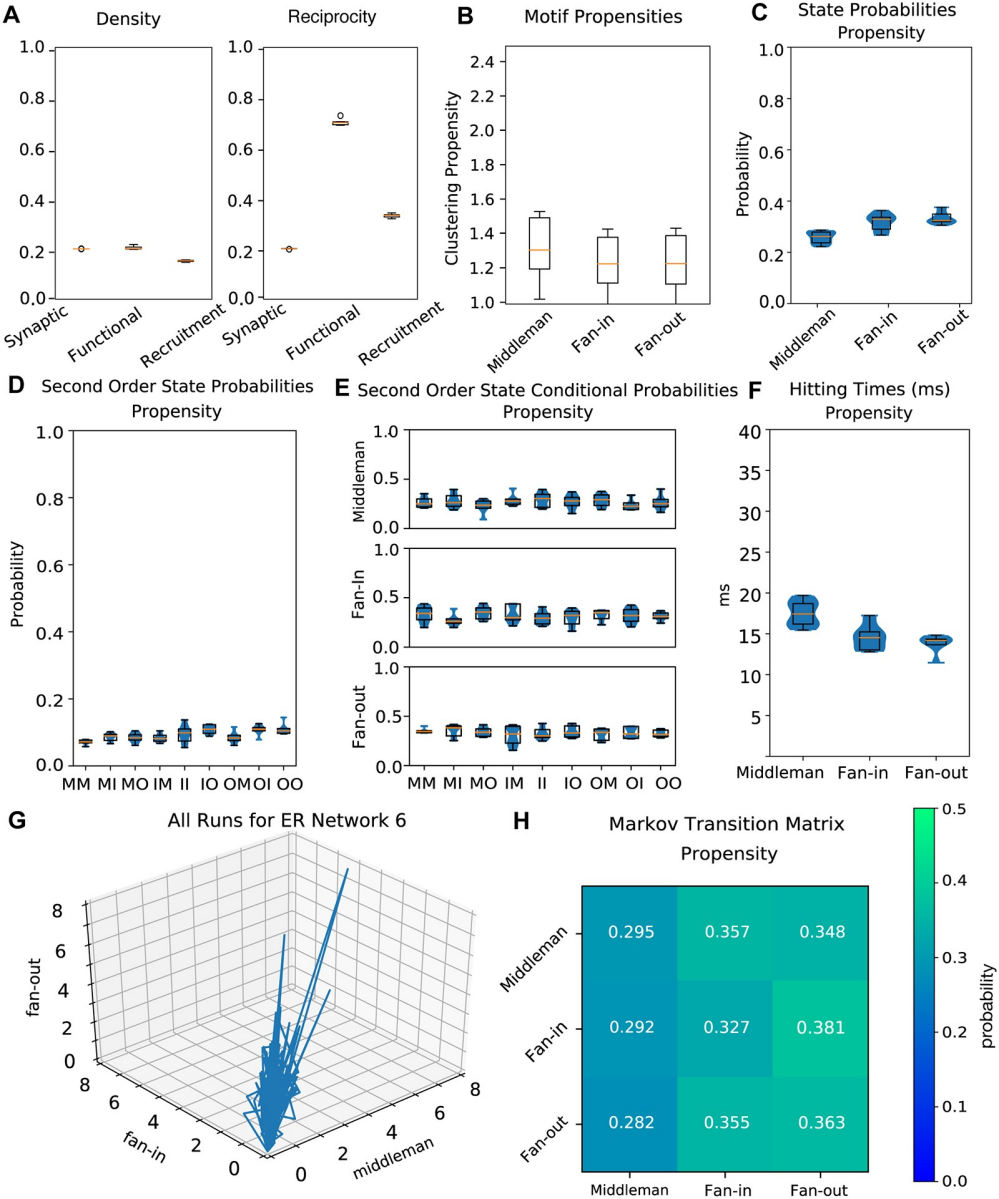
Unclustered (Erdős-Renyi) networks. A: Left, density (ratio of existing to possible connections) for synaptic, functional, and recruitment ER graphs. Right, reciprocity (ratio of reciprocal to all existing connections) for synaptic, functional, and recruitment ER graphs. B: Clustering propensity for isomorphic triangle motifs on ER graph simulations. The y-axis is scaled to match that of Fig 7A (clustering propensities on original graphs) and Fig 9B (clustering propensities on graphs with 1.5 times increased weights). C: Probabilities of dominance of each triangle motif. The dominant motif at a time point is given by the maximum of mean middleman, mean fan-in, and mean fan-out across units. D: Second order motif state probabilities for progression of temporal recruitment graphs. E: Probabilities for each motif to follow a given second order motif. F: Hitting times for each state for the Markov process defined by motif transition probabilities. G: Trajectories of all runs on a sample ER network in 3-dimensional isomorphic motif space. All runs reached completion. H: Markov Matrix for transition probabilities between motifs.

The networks on which we performed all our analyses have excitatory clusters of units. To test whether our results, including the motif cycling phenomenon, are dependent on this structure, we next examined non-clustered Erdős-Renyi (ER) graphs with *p*_*i*→*e*_ = 0.25 and *p*_*e*→*i*_ = 0.35. ER graphs had the same *p*_*e*→*e*_ and *p*_*i*→*i*_ values as the clustered networks. We found that transitions between motif types were also present in the activity of sustained runs on ER networks (Fig 10). The relative increase in clustering in ER graphs when comparing synaptic to recruitment graphs is substantially greater than seen in our graphs with excitatory synaptic clusters. In the synaptic networks, triplet clustering coefficients average 0.11. However, this value increased to 0.20, 0.09, and 0.15 for fan-in, fan-out, and middleman motifs in the recruitment graphs. The propensity values for all isomorphic motifs were consistently lower than those in 1.5x networks, as well as original networks, with means centered at 1.25 (Fig 10B). We find that unclustered graphs and clustered graphs differ significantly in first and second-order state probabilities, state conditional probabilities, hitting times, and state transition probabilities (Fig 10C, 10D, 10E, 10F and 10H) (*p* ≤ 5.1 ⋅ 10^−12^, *n* = 111, *p* ≤ 0.00021, *n* = 111, *p* ≤ 0.00017, *n* = 106, *p* ≤ 1.1 ⋅ 10^−7^, *n* = 106, *p* ≤ 6.2⋅10^−8^, *n* = 111 respectively). As in the case with the increased weights however the qualitative cycling of motifs was present in sustained runs.

## Discussion

This work demonstrates that higher-order structure is crucial for sustained low-rate and asynchronous spiking in recurrent networks such as neocortex. Within the range of dynamics surveyed, we found that rate, criticality, and synchrony overlapped substantially between sustained and truncated runs, although high firing rates corresponded with short run times consistent with previous work [12]. Thus first order statistics did not cleanly partition the stability of asynchronous spiking in our models. Our subsequent analyses of higher order structure revealed that there are many ways for network activity to ‘fail’ and only one specific way to ‘succeed’. To succeed, spikes must traverse the synaptic network in a coordinated way, cycling iteratively between the global dominance of three triplet motifs. The transitions between fan-in, middleman, and fan-out motifs reveal the necessity of balance between distribution of output and convergence of input. The presence of these motifs in the recruitment graphs demonstrates the functional routing of activity through synaptic connections. When synapses become stronger and more reliable, overall triplet clustering decreases while the reliability of their transitions remains, demonstrating that these motifs tightly control synaptic cooperativity. Their presence is able to compensate for a prevalence of weak synaptic connections and to maintain the asynchronous spiking regime. Higher order motifs in the recruitment network thus provide a direct link between asynchronous spiking and the stability of activity in that network.

Our study exclusively examined networks which produced low-rate, critical, and asynchronous spiking, consistent with activity recorded in awake state in neocortex. As such, the cyclic motif transitions which support stability in this regime may not generalize to regimes with bursting or synchronous activity. The dynamical features of neocortex are undoubtedly interrelated with the stability of those dynamics. However, given the prevalence of this spiking regime, our results provide an explanation for the prominence of higher order motifs in real data. Elevated motif counts have been observed in synaptic connectivity and in recordings of clustered activity in vivo [2, 3, 8, 22–28]. Through mechanisms of learning in neocortex such as STDP, functional patterns may be further strengthened to enhance integration in cortex. We wish to draw attention to the fact that our study focused on the whole-network scale. Individual units spiked only sparsely, making it difficult to continuously track single-unit motifs across small epochs of time since interspike intervals were generally larger than the intervals we analyzed. Regardless, functional networks summarizing long recordings from neocortex also report the prevalence of these motifs, albeit without this dynamic component. The models we used were constructed to simulate neocortex. The network structures we employed closely match experimental observations [2, 3] and the model units capture many of the statistics of neocortical neurons [5]. Our results provide, first and foremost, an account of at least one of the roles of beyond-pairwise interactions in the brain. Yet the behavior of these models may reflect necessary features of weakly-connected networks in which integration from multiple sources is necessary for the system to succeed. In such systems it is likely that stability relies on higher-order patterns. For example, the spread of rumours in a social network relies on integrating interactions. Social networks are small-world networks characterized by clusters, a feature which is present in our model as well as many other systems [1, 52]. The “illusion-of-truth” effect in rumour spreading on a social network has the integrate-and-fire property, where an individual may need to hear a rumour from multiple sources before they reach a confidence threshold to repeat it to others [53].

The necessity of higher-order patterns for stable asynchronous activity has strong implications for neural coding. Previous work has already demonstrated that correlations enhance coding, with triplet correlations having an advantage over pairwise, as well as the limited role of motifs larger than three nodes [5, 26, 28, 31–33, 54–57]. The neural code must rest upon a foundation of the maintenance of spiking, which we have shown in turn rests on higher-order motifs and coordinated synaptic integration in the awake dynamical state. Any two spikes must take place within some time interval for them to interact. The asynchronous and critical regime observed in vivo and in our models pushes the limits on what constitutes a cooperative event. In our model, the precise conditions for integration are dictated by the time constants we chose, while in neocortex the same time constants may vary and span some range. Neuromodulation, cognitive state, and a variety of other factors all dictate the requirements which need to be met for integration. Local connectivity certainly plays a large role as well. Consequently, the role of higher order interactions in coding and in coordinating synaptic integration may vary by brain region and state.

## Materials and methods

### Network structure

Our graphs are recurrent and sparsely connected networks of several thousand adaptive exponential leaky integrate-and-fire (AdEx) units with an extra poisson input term [37]. Synapses between all units are conductance-based. This enhances realism by taking neuron-specific state features into account during synaptic integration [37]. Specifically we define our neuron Voltage, *V*, as.

$$\begin{array}{c} C\frac{dV}{dt}=-g_{l}\Delta_{t}\text{exp}\left(\frac{V-V_{T}}{\Delta_{T}}\right)-g_{e}\left(V-E_{e}\right)-g_{i}\left(V-E_{i}\right)-g_{p}\left(V-E_{e}\right)-w \end{array}$$ (1)

adaptation current, *w*, as

$$\begin{array}{c} \tau_{w}\frac{dw}{dt}=a\left(V-E_{l}\right)-w \end{array}$$ (2)

excitatory conductance, *g*_*e*_, as

$$\begin{array}{c} \tau_{e}\frac{dg_{e}}{dt}=-g_{e} \end{array}$$ (3)

inhibitory conductance, *g*_*i*_, as

$$\begin{array}{c} \tau_{i}\frac{dg_{i}}{dt}=-g_{i} \end{array}$$ (4)

poisson input conductance, *g*_*p*_, as

$$\begin{array}{c} \tau_{p}\frac{dg_{p}}{dt}=-g_{p} \end{array}$$ (5)

A spike was said to occur if *V* > *V*_*t*_, after which *V* was set to *EL*, *w* was incremented by *b* and *g*_*e*_ and *g*_*i*_ were incremented by synapse weight if downstream of the spiking neuron.

For information on parameters, see S1 Table. Each network is comprised of 1000 inhibitory and 4000 excitatory units. Precise wiring probabilities between excitatory and inhibitory populations were determined through grid search within biological constraints.

Network synaptic connectivity is heterogeneously clustered [8]. For each network we defined 50 total clusters, with each excitatory unit randomly assigned to two clusters. Clusters thus vary in size and follow a binomial distribution. The wiring probability between two units within the same cluster is twice that of units in different clusters. Network cluster sizes range from 111 to 207 excitatory units (mean = 158.40, std = 12.27). Inhibitory units are not clustered; their wiring probability is uniform across the graph.

Edge weights follow a heavy-tailed distribution (Fig 1B). Edge weights that originate from inhibitory units have conductances which are ten times greater than those which originate from excitatory units, in accordance with experimental results [12].

### Network simulation

Each simulation was recorded at 0.1-ms temporal resolution. A trial began with 30 ms of Poisson input stimulus onto 500 randomly chosen units. After 30 ms the stimulus would cease and activity would propagate naturally through the network. The simulation would continue for as long as spiking activity is sustained, up to a maximum of 1 second. If during a simulation no spikes occur across the network for 100 ms, the network is deemed inactive and the simulation trial is halted. We found that all network simulations which reached 1 second were also able to sustain activity up to 10 seconds. We therefore chose one second as the marker for a network’s ability to sustain activity indefinitely, and as the definition of a successful run. Upon completion each simulation yields an output raster of spike times for every unit in the network. The Poisson input train, input units, network topology, and initial conditions of all units were recorded for each simulation. This enabled subsequent analyses and also allowed for re-use of a synaptic graph or re-instantiation of a simulation using some of the original settings while varying others.

### Parameterization

Our models are constructed to parallel the features of biological neural networks, but are also constrained by considerations of computing resources. In a study which modeled cortex with high biophysical and anatomical detail, simplifying the neuron model did not lead to drastic differences in the network’s behavior from the detailed model or from in vivo results. Most qualities remained unchanged, suggesting that in many cases extremely granular models are not necessary to yield experimental insights [37]. Instead, the most important feature for retaining qualitative correspondence are the rules of synaptic connectivity. Therefore we required our models’ connectivity parameters to closely match those of biological neural networks.

The probabilities of wiring between excitatory (E) and inhibitory (I) populations in our models were taken directly from or bounded by the results of biological experiments. The wiring probabilities from E to other E units and from I to other I units in neocortex are well-studied, but there is less data on connections from E to I and from I to E. We therefore used an algorithmic approach to find the optimal values. Beginning within a biological range, we used grid search to find values of *p*_*e*→*i*_ and *p*_*i*→*e*_ that led to successful maintenance of activity at the lowest possible rates. We used these optimal wiring rules to construct all synaptic graphs in this study.

Two iterations of grid search were used to find the wiring parameters needed to maintain naturalistic spiking for the duration of a simulation (Fig 2A). We searched for the optimal probability of connection from excitatory to inhibitory units, *p*_*e*→*i*_, and the optimal probability of connection from inhibitory to excitatory units, *p*_*i*→*e*_, such that networks would sustain activity at the lowest possible rates. In the first iteration, we used a low resolution grid (space size 0.001) to search for *p*_*e*→*i*_ within the range 0.16 to 0.24 and *p*_*i*→*e*_ within the range 0.29 to 0.37. These two ranges were taken from known wiring probabilities in neocortex. Each grid space was visited ten times to achieve an average measure of rate and completion. This isolated a region of interest where the rate was lowest, between *p*_*e*→*i*_ values of 0.210 and 0.230, and between *p*_*i*→*e*_ values of 0.300 and 0.320. We used a higher resolution grid (space size 0.0001) to explore this region.

For all subsequent simulations we used the best results obtained from grid search. The optimal probability of wiring for excitatory to inhibitory units, *p*_*e*→*i*_, was found to be 0.22, and the optimal value for *p*_*i*→*e*_ was 0.31. The values for *p*_*e*→*e*_ and *p*_*i*→*i*_ were taken from known wiring probabilities in neocortex, and were 0.20 and 0.30 respectively [29]. Based on these wiring rules, we constructed synaptic graphs of networks for simulations. Each synaptic graph is a matrix *W* where the value in *w*_*ij*_ denotes the weight of the directed connection from unit *i* to unit *j*.

### Scores

To evaluate the biological realism of constructed networks, we computed several measures of network activity for both excitatory and inhibitory subpopulations. All measures were calculated based on spiking activity between 30ms after trial start and 50ms prior to trial truncation. Networks were evaluated on rate, defined as average spike frequency over the course of each trial. Networks were also evaluated on branching parameter as a measure of network criticality [13]. A branching value of 1 indicates that for every ‘ancestor’ unit that is active, there is an equal number of ‘descendant’ units active at the next time step. On average, the number of units active over the course of a trial in a critical network stays constant.

Branching was mathematically defined as:

$$\begin{array}{c} \sigma =\sum_{d=0}^{n_{max}}d\cdot p\left(d\right), \end{array}$$ (6)

$$\begin{array}{c} p\left(d\right)=\sum_{avalanches}(\frac{n_{\Sigma a|d}}{{}_{\Sigma n_{a}}})(\frac{n_{max}-1}{n_{max}-n_{a}}) \end{array}$$ (7)

where *σ* is the branching parameter, *d* is the number of descendants, *n*_*max*_ is the maximum number of active neurons, *n*_*a*_ is the number of ancestor neurons, *n*_*d*_ is the number of descendant neurons, *n*_Σ*a*|*d*_ is the number of ancestor neurons in all avalanche events that involved d descendants, and *n*_Σ*a*_ is the total number of neurons involved in avalanches. The branching parameter describes the network as a whole; it cannot be calculated for isolated units. For a given simulation, we calculated network branching at discrete, sequential time steps throughout. We used the same temporal resolution (5 ms) as used for determining the functional graph; all spikes at time *t* are ancestors, and all spikes from *t* + 5 to *t* + 20 ms are descendants. We then averaged the network branching parameter across all time steps to get the overall branching score for that simulation. Networks were further evaluated on their level of asynchrony, since biological networks display asynchronous activity. In order to evaluate asynchrony rapidly enough to make grid search feasible, a heuristic for synchrony was computed as the variance of mean voltage normalized by the mean variance of each neuron. An upper threshold of 0.5 was considered appropriate for network asynchrony. The threshold was evaluated empirically by examining a population of inhomogeneous Poisson neurons with underlying Gaussian firing rates where covariance across the underlying Gaussian processes was the varied parameter. The Van Rossum spike distance [45] was used as the measure of asynchrony for all analyses outside of initial grid search. The Van Rossum spike distance was calculated as follows: each spike train was convolved with an exponential kernel with time constant *τ* = 10 ms, we then took the distance to be the mean L2 norm between the resulting traces normalized by $\sqrt{\frac{1}{\tau }}$.

### Triplet motifs

The clustering coefficients for the four triplet motifs are calculated in the following manner [49].

Let *t*_*i*_ denote the actual number of triplets of a motif type in the neighborhood of unit *i*, and *T*_*i*_ denote the maximum number of such triplets that unit i could form. We will build intuition by beginning with the case of a binary directed graph, or an unweighted connectivity matrix. Let *A* represent this graph, with *a*_*ij*_ = 1 indicating the presence of a directed connection from node *i* to node *j*. Raising the matrix *A* to the nth power yields the number of paths of length *n* between nodes *i* and *j*.

Let us first consider the cycle motif; in order for unit *i* to participate in a cycle, it must have an edge directed to a second unit, that second unit must have an edge directed to a third unit, and that third unit must have an edge pointing back to unit *i*. The path length is 3, and it both begins and ends at unit *i*. Thus we calculate *A*^3^ and extract the values along the diagonal, or $A_{ii}^{3}$. This gives the number of actual cycle motifs unit *i* forms.

Counts of the three isomorphic motifs are calculated in a similar way, but they require the additional involvement of *A*^*T*^. Taking the transpose of graph *A* reverses the directionality, so that connections from *i* to *j* are now those from *j* to *i*. We would like to trace a path of length 3 from i back to i to form an isomorphic triangle, but exactly one of the steps must be against the true direction of that edge (Fig 5). Beginning with a middleman reference node, the first step is ‘with the flow’, the second step is invariably ‘against the flow’, and the final step back to i is again ‘with the flow’. Therefore *AA*^*T*^ *A*_*ii*_ gives the number of actual middleman motifs unit i forms. Since fan-in and fan-out motifs are isomorphic to middleman by rotation, we simply rotate which step is ‘against the flow’ to yield the count of fan-in and fan-out motifs. The number of actual fan-in motifs unit i forms is $A^{T}A_{ii}^{2}$, and the number of fan-out motifs is $A^{2}A_{ii}^{T}$.

Now that we can calculate the actual counts, the possible counts of each motif *T*_*i*_ are easily intuited as a combinatorics problem. Let us begin again with the cycle motif. To form a cycle, node i requires one edge directed towards it and one edge directed away from it. The number of possible pairs of in and out edges from node *i* is calculated by multiplying the out-degree of node *i* with the in-degree of node *i*. In-degree and out-degree refer simply to the number of edges that are directed in or out of a given node. Some edges may be bidirectional—these cannot be part of a true cycle motif. The number of bidirectional edges is subtracted from the product of in- and out-degrees. The final *T*_*i*_ for the cycle motif is

$$\begin{array}{c} T_{i}=d_{i}^{in}d_{i}^{out}-d_{i}^{↔} \end{array}$$ (8)

The Ti for middleman is in fact equal to that for cycle, since forming a middleman has the same requirements—one edge directed inward paired with one edge directed outward.

A fan-in motif requires two edges directed in towards the reference node. There are $d_{i}^{in}$ number of choices for the first inward edge. Once that choice has been made, there are $d_{i}^{in}-1$ choices remaining for the second inward edge. Thus we multiply the two to yield *T*_*i*_ for the fan-in motif.

$$\begin{array}{c} T_{i}=d_{i}^{in}\left(d_{i}^{in}-1\right) \end{array}$$ (9)

Fan-out is similar—we simply substitute in-degrees with out-degrees since a fan-out motif requires two edges directed out from the reference node. Ti for the fan-out motif is thus

$$\begin{array}{c} T_{i}=d_{i}^{out}\left(d_{i}^{out}-1\right) \end{array}$$ (10)

Now that we have both the actual and possible counts for each motif type, the triplet clustering coefficients of node i are simply their ratios. That is,

$$\begin{array}{c} C_{i}^{⋆}=\frac{t_{i}^{⋆}}{T_{i}^{⋆}} \end{array}$$ (11)

If we were interested in binary graphs, we would end here. However, our graphs of interest have weights associated with each directed edge. There are multiple ways to account for edge weights when calculating clustering coefficients. One way is to consider only the weights of the two edges that are incident to reference node *i*. Alternatively, the weights of all three edges in a triplet can be taken into consideration. The latter is the chosen method, since we desire a measure of central tendency. The total contribution of a triplet to the clustering coefficient is thus the geometric mean of its weights.

Let *W* denote our weighted directed graph. For a triplet in this graph with edge weights *w*_*ij*_, *w*_*ih*_, and *w*_*jh*_, the geometric mean is ($w_{ij}\cdot w_{ih}\cdot w_{jh}{)}^{\frac{1}{3}}$. We can extend this to the entire graph by, Instead of using a binary graph as matrix *A* in the calculation of *t*_*i*_, using $A=W^{\frac{1}{3}}$, which is the matrix that results from taking the cubic root of every entry in *W*. We also note that this formulation is invariant to the choice of reference node in a triplet. Incorporating weights only modifies the value of *t*_*i*_. It remains a measure of the actual triplets present—instead of counts it is now a weighted measure. The denominator *T*_*i*_ still refers to maximum possible counts. It follows that the clustering coefficient for node *i* can only be 1 (maximum) when its neighborhood truly contains all triplets that could possibly be formed and every edge in each triplet is at unit (maximum) weight. The complete clustering coefficient formulas of weighted directed graphs are given in Fig 5.

### Active subgraphs

For any small span of time in a trial, only a subset of all units in the graph will be active. The subset of units which spike in some defined time window form the active subgraph for that time window. We binned spikes into a temporal resolution of 10 ms, so that each complete 1-second simulation resulted in 99 time bins. For each time bin t we defined an active subgraph. If a unit spiked within time bin t, that unit will be part of the active subgraph for time bin t. All units which did not spike within that particular time bin are not included in that particular active subgraph. Since there are 99 time bins for a complete 1-second simulation, there are also 99 active subgraphs in sequence. Edge weights between units in an active subgraph are equal to those from the corresponding edges (between active units) in the ground truth synaptic graph.

### Functional subgraphs

We calculated motifs in the underlying synaptic graphs and found that all four clustering coefficients were equivalent when averaged across each graph, as expected.

To apply motif analysis to activity, we needed to infer functional graphs from spiking activity to summarize network dynamics. Directed edge weights in a functional graph represent the likelihood of a functional relationship in the activity between every pair of units.

We used mutual information (MI) to infer functional graphs from all spikes across the course of a trial, regardless of the trial’s duration (complete or truncated). This results in a single functional graph for each trial. We chose to perform functional inference using the full spike set because this yields functional graphs with higher fidelity and greater sparsity.

The MI method we used is the confluent mutual information between spikes. At a conceptual level, an edge inferred from unit i to unit j using confluent MI means that unit j tends to spike either in the same time bin or one time bin after unit i spikes. Since spikes are binned at 10ms resolution, this method encompasses a delay of 0 to 20 ms. This delay is appropriate because we found that presynaptic spikes yielded a maximal response from all postsynaptic neurons at a delay of 5 to 20 ms.

Mathematically, we defined an indicator function on the spike train of neuron *j*, *s*(*j*) evaluating to 1 in the case where there is a spike at time *t* or *t* + 1, an indicator function on the spike train of neuron *i*, *t*(*j*), evaluating to 1 in the case where there is a spike at time *t*, and considered the mutual information between them. The resulting networks were further processed by removing weights corresponding to neurons with negative pairwise correlations. Networks were then re-expressed to minimize skewness, and background signals were removed by accounting for background signal and considering weights as the residual resulting from linear regression on background strength. Finally we considered the z-normalized residual graph to account for heteroskedasticity [34]. This yields weighted values, for which we establish 0 as a threshold. All positive normed residual MI values are included in the full functional graph.

### Recruitment graphs

The recruitment graph represents both the activity and the underlying connections of a network. A recruitment graph is defined separately for each 10 ms time bin of a given trial, thus yielding a temporal sequence of graphs. Each graph is calculated as the intersection of the functional graph, which is unique to every trial, and the active subgraph, which is unique to every 10 ms time bin. All edges in the recruitment graph come from underlying synaptic wiring, contained in the active subgraph, while edge weight values come from the inferred functional graph. In other words, for all edges *i* → *j* where *w*_*functional*,*ij*_ > 0 in the confluent MI functional graph and *w*_*synaptic*,*ij*_ > 0 in the active subgraph of time bin *t*, the edge in the recruitment graph for time bin t takes on the value of *w*_*functional*,*ij*_. All other recruitment graph edges have value 0.

Just like the sequence of active subgraphs, there are 99 sequential recruitment graphs at 10 ms temporal resolution for every complete 1-second simulation trial. Triplet clustering coefficients were calculated for every unit on each 10 ms recruitment graph, then averaged across the population to yield the whole-network clustering coefficients for that 10 ms time window. These methods allow us to observe how motif clustering changes in the recruitment graphs across time.

### Clustering propensity

Networks may have very different connection densities, which would impact motif clustering coefficients. This is especially true as the active subnetwork changes in time, and for ER networks in comparison to clustered model graphs. We therefore used weighted and unweighted clustering propensity which enables meaningful comparisons between networks with different connection densities.

Our measure of weighted propensity begins with calculating the triplet motif clustering coefficients for each unit in the recruitment graph of every time bin. Then, for each time bin t we generate ten simulated graphs. These graphs have the same edges as the original recruitment graph at time *t*, with edge weights randomly sampled from the underlying distribution of functional edge weights. Motif clustering coefficients are calculated for units in each of the simulated graphs, then averaged for each unit and each motif type. The clustering coefficients of the units in the original graph at time *t* are normalized by the average of the ten simulated graphs’ clustering coefficients, yielding the unit-wise clustering propensity at time *t* for each triplet motif. We used these values to perform all unit-wise motif analysis. In order to examine motifs at a whole-network level, for each motif type at time *t* we average across all units with nonzero clustering propensity values for that motif type.

Unweighted propensity is calculated similarly, considering the functional networks’ unweighted directed clustering to that expected in both an ER graph as well as a small world graph. Thus, in addition to controlling for density, weighted propensity also measures the extent to which the specific edge weights in the recruitment graph impact triplet motif clustering, while unweighted propensity measures the same for specific structure of the recruitment graph. A weighted propensity of 1 indicates that specific edge weights play a negligible role in clustering, since random edge weights would still yield the same clustering coefficients, while an unweighted propensity of 1 indicates that the specific structure of the network is not important for clustering.

### Erdős-Renyi graph simulations

Unclustered ER Graph simulations were performed using networks consisting of 1000 excitatory neurons, 200 inhibitory neurons and 50 Poisson input neurons. These populations were connected with *p*_*ee*_ = 0.2, *p*_*ii*_ = 0.3, *p*_*ie*_ = 0.25 and *p*_*ei*_ = 0.35. Synaptic weights relative to leak conductance were drawn from a log normal distribution (mean = 0.60, variance = 0.11), with i to e connections scaled up 50% [29].

### Overlap index

Overlap Index was used to measure the degree of overlap between two probability distributions. It is defined as $O=\sum_{i}\text{min}p_{i1},p_{i2}$, where *i* is histogram bin index. If two distributions do not overlap at all they will have an overlap index of 0, if they are identical they will have an overlap index of 1.

### Probability vectors

To quantify the cyclic transitions between relative prominence of motifs over time, we examined the dominant motif of the network for a given recruitment graph. We define the dominant motif of a graph as the maximum of the demeaned propensities for middleman, fan-in, and fan-out. We consider the demeaned values of each motif in order to account for the different relative magnitudes of motifs without affecting scaling in the cycle structure. Examining first order probabilities, which is the probability of a motif dominating a recruitment graph in a given run, on the time series of recruitment graphs from sustained and truncated runs shows that there is a significant difference between the distributions defining these values across each type of run.

To further characterize the transitions between different dominant motifs we fit a Markov model to the series of dominant motifs across recruitment graphs in both truncated and sustained networks. We again find a significant different (*p* < 1⋅10^−15^, *n* = 884 for all markov parameters). This all suggests that the failure to propagate found in some networks is tied to the inability to recruit the cyclic structure that we find to be a hallmark of sustained activity.

### Statistical testing: Two-sample chi squared test

P values for comparisons between distributions of different types of network activity were done by two sample chi square test.

## Supporting information

S1 Table Neuron parameters. Parameters used for simulation of adaptive exponential integrate and fire neurons.
(PDF)
