# Supplementary material for: Cyclic transitions between higher order motifs underlie sustained asynchronous spiking in sparse recurrent networks
Source: PLoS Comput Biol. 2020 Sep 30;16(9):e1007409. doi: 10.1371/journal.pcbi.1007409 (PMC7549833; doi:10.1371/journal.pcbi.1007409)
Supplement: S1 Table — Parameters used for simulation of adaptive exponential integrate and fire neurons. (PDF) [file pcbi.1007409.s001.pdf]

| Parameter | Name | Value |
|---|---|---|
| $C$ | membrane capacitence | 281 pF |
| $g_L$ | leak conductance | 30 nS |
| $E_L$ | leak reversal potential | -70.6 mV |
| $E_E$ | excitatory reversal potential | 0 mV |
| $E_I$ | inhibitory reversal potential | -75 mV |
| $V_T$ | spike threshold | -50.4 mV |
| $\Delta_T$ | slope factor | 2 mV |
| $\tau_w$ | adaptation time constant | 144 ms |
| $\tau_e$ | excitatory time constant | 10 ms |
| $\tau_i$ | inhibitory time constant | 3 ms |
| $\tau_p$ | poisson time constant | 3ms |
| $a$ | subthreshold adaptation | 4 nS |
| $b$ | spike triggered adaptation | .0805 nA |

**S1 Table.**
